# Cell proliferation detected using [^18^F]FLT PET/CT as an early marker of abdominal aortic aneurysm

**DOI:** 10.1007/s12350-019-01946-y

**Published:** 2019-11-18

**Authors:** Richa Gandhi, Christopher Cawthorne, Lucinda J. L. Craggs, John D. Wright, Juozas Domarkas, Ping He, Joanna Koch-Paszkowski, Michael Shires, Andrew F. Scarsbrook, Stephen J. Archibald, Charalampos Tsoumpas, Marc A. Bailey

**Affiliations:** 1 Leeds Institute of Cardiovascular and Metabolic Medicine, School of Medicine, University of Leeds, 8.49c Worsley Building, Clarendon Way, Leeds, LS2 9NL United Kingdom; 2 Institute of Medical and Biological Engineering, School of Mechanical Engineering, University of Leeds, Leeds, United Kingdom; 3 Department of Biomedical Science, PET Research Centre, University of Hull, Hull, United Kingdom; 4 Nuclear Medicine and Molecular Imaging, Department of Imaging and Pathology, KU Leuven, Leuven, Belgium; 5 Experimental & PreClinical Imaging Facility (ePIC), School of Medicine, University of Leeds, Leeds, United Kingdom; 6 Leeds Institute of Medical Research at St James’s, University of Leeds, Leeds, United Kingdom; 7 Biomedical Engineering and Imaging Institute, Icahn School of Medicine at Mount Sinai, New York, NY USA; 8 Invicro, London, United Kingdom; 9 The Leeds Vascular Institute, Leeds General Infirmary, Great George Street, Leeds, United Kingdom

**Keywords:** Vascular biology, aneurysms, PET, molecular imaging, vascular imaging, pre-clinical imaging

## Abstract

**Background:**

Abdominal aortic aneurysm (AAA) is a focal aortic dilatation progressing towards rupture. Non-invasive AAA-associated cell proliferation biomarkers are not yet established. We investigated the feasibility of the cell proliferation radiotracer, fluorine-18-fluorothymidine ([^18^F]FLT) with positron emission tomography/computed tomography (PET/CT) in a progressive pre-clinical AAA model (angiotensin II, AngII infusion).

**Methods and Results:**

Fourteen-week-old apolipoprotein E-knockout (ApoE^−/−^) mice received saline or AngII via osmotic mini-pumps for 14 (*n *= 7 and 5, respectively) or 28 (*n *= 3 and 4, respectively) days and underwent 90-minute dynamic [^18^F]FLT PET/CT. Organs were harvested from independent cohorts for gamma counting, ultrasound scanning, and western blotting. [^18^F]FLT uptake was significantly greater in 14- (*n *= 5) and 28-day (*n *= 3) AAA than in saline control aortae (*n *= 5) (*P *< 0.001), which reduced between days 14 and 28. Whole-organ gamma counting confirmed greater [^18^F]FLT uptake in 14-day AAA (*n *= 9) compared to saline-infused aortae (*n *= 4) (*P *< 0.05), correlating positively with aortic volume (*r *= 0.71, *P *< 0.01). Fourteen-day AAA tissue showed increased expression of thymidine kinase-1, equilibrative nucleoside transporter (ENT)-1, ENT-2, concentrative nucleoside transporter (CNT)-1, and CNT-3 than 28-day AAA and saline control tissues (*n *= 3 each) (all *P *< 0.001).

**Conclusions:**

[^18^F]FLT uptake is increased during the active growth phase of the AAA model compared to saline control mice and late-stage AAA.

**Electronic supplementary material:**

The online version of this article (10.1007/s12350-019-01946-y) contains supplementary material, which is available to authorized users.

## Introduction

Abdominal aortic aneurysm (AAA) is a focal dilatation of the abdominal aorta that progresses to rupture, which confers high mortality.^1^ AAA is detected incidentally or through ultrasound scanning (USS)-based screening programmes.^2^,^3^ Once detected, USS monitoring is used to track AAA diameter until it reaches the 5.5-cm intervention threshold; this can take > 10 years and places a discernible burden on patients.^4^,^5^ A non-invasive stratification biomarker applied at the time of AAA detection to personalize monitoring regimens and identify those who may benefit from a specific medical therapy or early intervention would be beneficial. Positron emission tomography (PET) can provide information about the molecular processes beyond the anatomical characteristics of USS or computed tomography (CT) and may therefore be useful for this purpose.

Fluorine-18 fluorodeoxyglucose ([^18^F]FDG) is a glucose uptake marker commonly used in oncology as an indicator of high metabolic activity, such as at sites of inflammation, and is the most commonly administered PET radiotracer in clinical practice. The data on [^18^F]FDG uptake in AAA are mixed and conflicting.^6^-^9^ Some studies suggest that increased [^18^F]FDG uptake positively correlates with AAA progression,^10^-^12^, while others indicate an inverse relationship or lack of correlation between [^18^F]FDG uptake and aortic size.^13^,^14^ The Sodium Fluoride Imaging of AAA (SoFIA3) trial recently demonstrated that fluorine-18 sodium fluoride AAA uptake (reflecting microcalcification) predicts AAA progression and rupture, providing the first proof-of-concept data for PET/CT as a AAA stratification biomarker 15; however, the mechanism remains unclear.

Maegdefessel et al. showed that modulating microRNA-21 expression reduces cell proliferation, protecting mice from AAA expansion.^16^ Smooth muscle cell de-differentiation and proliferation is an early event in AAA.^17^ Preventing this event by deleting Kruppel-like factor-4 prevents AAA formation in mice.^18^ These data point to a period of pathological cellular remodeling in early AAA and suggest that anti-proliferative therapy might be beneficial. Therefore, detecting cell proliferation in vivo may be useful. Our hypothesis is that this proliferative remodeling is detectable using PET/CT with an analogue of the pyrimidine deoxynucleoside thymidine, fluorine-18 fluorothymidine ([^18^F]FLT). This tracer accumulates in proliferating cells and reflects thymidine kinase-1 (TK-1) activity. Here, we investigate [^18^F]FLT as a PET/CT radiotracer in the progressive angiotensin II (AngII) infusion pre-clinical model of AAA.

## Methods

### Animals

All animal work was conducted in accordance with the UK Home Office, Animals (Scientific Procedures) Act 1986 under Project Licence P606320FB. Male Jax^TM^ apolipoprotein E-knockout mice (ApoE^−/−^; B6.129P2-Apoetm1Unc/J; Charles River, UK) were used for experiments at 14 weeks of age. Male C57BL6/J mice (Charles River, UK) were used at 8 weeks of age for baseline biodistribution studies. All animals were part of the Jackson Laboratories Genetic Stability Programme to limit cumulative genetic drift. Mice were maintained at 21 °C with a 12-hour light/dark cycle and 50% to 70% humidity in GM500 individually ventilated cages (Techniplast, Italy) and fed standard chow diet and Hydropac water *ad libitum.* All mice were provided with a housing dome and two chew sticks as environmental enrichment.

For the AngII AAA model, 14-week-old male ApoE^−/−^ mice (Jackson Laboratories) received human AngII (Sigma A9525) infusions at 750 ng·kg^−1^·min^−1^ via Alzet^®^ 1002 (14 days) or 1004 (28 days) osmotic mini-pumps that were implanted subcutaneously by posterior neck incision under recovery isoflurane anaesthesia, as described previously.^19^ ApoE^−/−^ controls received saline infusions via identical pumps.

### [^18^F]FLT Production

[^18^F]FLT was prepared from fluorine-18 fluoride produced using an on-site 7.5-MeV ABT Biomarker Generator cyclotron and purified using an in-house-developed microfluidic electrochemical cell for electrode trapping, through which irradiated target water (0.5 mL, oxygen-18 H_2_O) containing ca. 1 GBq (27.03 mCi) of fluorine-18 fluoride was pumped at 0.2 mL·min^−1^ while applying a 20 V potential, and the cell was flushed with 2 mL of MeCN at 1 mL·min^−1^ with no potential applied. Subsequently, 0.4 mL of a solution containing KHCO_3_ (30 mM) and K222 (37 mM) in MeCN was pumped through the cell at 40°C at 0.1 mL·min^−1^. Ten mg of fluorothymidine precursor 3′-N-Boc-5′-dimethoxytrityl-3′-O-nosyl-thymidine was added to the released solution to perform a radio-labeling reaction at 100 °C for 10 minutes. After the fluorination, the unreacted fluorine-18 fluoride was trapped on the neutral alumina cartridge (light) and reaction mixture treated for 5 minutes at room temperature with an equivalent volume of 2 N HCl solution. The mixture was then neutralized with a stoichiometric amount of 8 N NaOH solution and purified by semi-preparative high-performance liquid chromatography on an ACE 5 C18 10 × 250 5A column eluted with 35% acetonitrile in water (both 0.1% trifluoroacetic acid) (flow rate= 4.7 mL·min^−1^, *R*_t_= 12 minutes). The high-performance liquid chromatography fraction containing partially protected product was diluted two to three-fold with water and passed over an HBL Oasis C18 cartridge. Trapped product was eluted with 0.5 mL of ethanol and 3 mL of diethyl ether and dried at 60 °C under an inert gas stream. The heat applied for drying generated the fully deprotected [^18^F]FLT (confirmed by analytical high-performance liquid chromatography with a cold fluorine-19 FLT standard), which was re-dissolved in 10% ethanol/phosphate-buffered saline solution, filtered through a 0.22-µm filter for sterility, and delivered for animal administration. Tracer preparation started with 1.0 to 1.3 GBq (27.03 to 35.14 mCi) of cyclotron-produced fluorine-18 fluoride and yielded 40 ± 8 MBq (1.08 ± 0.22 mCi) (n.d.c.) [^18^F]FLT intravenous-injectable formulation in 136 ± 15 minutes (*n *= 5) (RCY (decay-corrected)= 8% ± 2%).

### In Vivo PET/CT

Mice were induced with 5% isoflurane/oxygen (v/v) anaesthesia before maintenance at 2% at 1 L·min^−1^. Mice were cannulated in the tail vein using a bespoke catheter before being placed into an imaging cell where temperature and respiration were monitored (Minerve, France). [^18^F]FLT was injected with the following means ± standard deviations (SDs) of activity in 200 µL of 0.9% saline solution (Aqupharm No1, Animalcare Ltd., York, UK) through the pre-cannulated lateral tail vein at the beginning of a 90-minute dynamic imaging sequence: 7.27 ± 2.89 MBq (0.20 ± 0.08 mCi) (14-day scans; *n *= 12) and 10.08 ± 1.77 MBq (0.27 ± 0.05 mCi) (28-day scans; *n *= 7). Images were acquired using the Super Argus (Sedecal) small-animal PET/CT scanner, with animals placed prone and the field-of-view centered on the abdominal aorta. Mice were maintained at 1% anaesthesia during scanning, with temperature and respiration monitored throughout. Following the PET scan, a CT image was acquired for anatomic co-registration (40 kV, 140 µA, 360 projections, 8 shots). PET images were histogrammed into 15 2-seconds, 2 15-seconds, 4 60-seconds, 1 300-seconds, and 8 600-seconds frames and reconstructed using the three-dimensional ordered subsets expectation maximization algorithm with 2 iterations and 16 subsets with attenuation correction, yielding voxel dimensions of 0.39 × 0.39 × 0.78 mm^3^.

### PET/CT Image Analysis

Animals with failed or paravenous radiotracer injection (2/5 in the saline control group, 2/5 in the 14-day AngII AAA group, and 1/4 in the 28-day AngII AAA group) were excluded from the analysis. Three-dimensional isocontour regions of interest (ROIs) incorporating the supra-renal abdominal aortic area (between the renal arteries and diaphragm) were manually drawn on the reconstructed images using AMIDE v1.0.4 software. The aortic ROI was constructed between the kidneys and anterior to the anterior border of the vertebral column based on the CT images including the most prominent PET signal in the 80 to 90-minute timeframe. For saline controls, the aortic ROI was constructed between the kidneys, superior to the bladder, and anterior to the anterior border of the vertebral column based on the CT images. These ROIs had dimensions of 1.08 mm^3^ and contained 15 voxels (see Supplementary Figure 1 for example visual representation of ROIs). Incorporating mouse weights and injected radioactivity doses, the output statistics revealed the maximum and mean standardized uptake values (SUVs-) across all frames. These values were used to generate time-activity curves for ROIs in the abdominal aorta (as shown in Supplementary Figure 2), spleen, kidneys, and urinary bladder. The maximum SUV (SUV_max_) was chosen as the metric of choice to quantify the aortic ROI to avoid partial volume effects of the adjacent kidneys and bladder, where high [^18^F]FLT signals were observed consistently.

### Histological Staining

Murine aortae were fixed in situ by perfuse fixation with 10 mL of phosphate-buffered saline followed by 5 mL of 4% paraformaldehyde in terminally anaesthetized animals and then fixed at 4 °C for 48 hours before they were embedded in Cellwax (Cellpath Ltd., UK) using a Leica EG1150H embedding station. Four-µm thickness sections were cut onto Plus Frost slides (Solmedia, UK) using a Leica RM2235 microtome. Proliferating cells were stained with rabbit anti-mouse Ki67 antibody (ab15580, Abcam; 1:750 dilution) for 1 hour and Menapath Polymer HRP secondary antibody (Menarini Diagnostics Ltd., Winnersh, UK) for 30 minutes. Slides were counterstained with haematoxylin and imaged using the Aperio^®^ AT2 (Leica Biosystems, Wetzlar, Germany) digital pathology slide scanner with × 20 maximal magnification. All quantitative analyses were performed using ImageJ 1.51k software.^20^ For quantification of the Ki67 staining, the proportion of Ki67-positive nuclei was determined in three 50-µm^2^ ROIs and averaged for each animal.

### Protein Quantification

An independent cohort of animals was used to harvest tissue at baseline and after 28-day saline, 14-day AngII, and 28-day AngII infusions. Aortic tissue (supra-renal abdominal aortic segment only) was harvested under terminal isoflurane anaesthesia, snap-frozen in liquid nitrogen after phosphate-buffered saline perfusion, and stored at − 80 °C until use. Proteins were extracted using cell extraction buffer (FNN0011, Invitrogen) supplemented with Protease Inhibitor Cocktail (P8340, Sigma). The DC Protein assay kit (5000112, Bio-Rad) was used to quantify isolated proteins. The average yield was more than 3 mg of protein per aorta. Western blotting was performed for TK-1, a key enzyme expressed during DNA synthesis and the substrate for [^18^F]FLT, and the specific carriers responsible for the transport of both [^18^F]FLT and thymidine across the cell membrane: equilibrative nucleoside transporter (ENT)-1 and -2 and concentrative nucleoside transporter (CNT)-1 and -3. For each protein, blots were conducted as per the manufacturer’s suggestions using 100 µg of protein per well. Antibodies and dilutions used were anti-TK-1, 1:500 (GTX113281; GeneTex, CA, USA); ENT-1, 1:1000 (ab135756); ENT-2, 1:1000 (ab181192); CNT-1, 1:500 (ab192438); CNT-3, 1:500 (ab223085); and β-actin, 1:1000 (Ab8226; all Abcam, Cambridge, UK). Proteins were detected using SuperSignal West Femto Maximum Sensitivity enhanced chemiluminescent substrate (ThermoFisher Scientific, Loughborough, UK). ImageJ 1.51k software was used to quantify the optical intensity of the bands relative to that of corresponding β-actin bands.^20^

### In Vivo USS

USS was performed under 5% recovery isoflurane anaesthesia before maintenance at 2% using the Vevo2100 high-resolution (30 µm), high-frequency pre-clinical µUSS system (Visualsonics, FUJIFILM VisualSonics, Inc., Toronto, ON, Canada) with an MS-550D transducer at a 40-MHz frequency on a heated platform maintaining a core body temperature of 35 to 37 °C. Prior to imaging, the abdomen area was shaved and hair removed using depilatory cream. Imaging was performed using Aquasonic^®^ clear gel (Parker Labs). Transverse imaging was acquired with a motor along an 11.96-mm ROI from the right renal artery in a cranial direction with 157 frames at 0.076-mm intervals gated for respiration with electrocardiographic triggering 50 ms after the *r*-wave. Images were reconstructed and measured using Vevo Lab v1.7.0 (VisualSonics, FUJIFILM VisualSonics, Inc., Toronto, ON, Canada) to generate three-dimensional lumen volume measurements. For the co-registration analysis, bony landmarks on CT and USS were aligned and displayed using ImageJ 1.51k.

### Ex Vivo Gamma Counting

Animals were anaesthetised with 5% isoflurane with an oxygen flow rate of 2 L·min^−1^. [^18^F]FLT in 100 µL of 0.9% saline solution (Aqupharm No1, Animalcare Ltd., York, UK) was injected into the tail vein at the following doses (mean ± SD): C57BL6/J study, 6.07 ± 2.57 MBq (0.16 ± 0.07 mCi); AngII AAA study, 0.25 ± 0.32 MBq (0.01 ± 0.01 mCi). For the AngII AAA ex vivo gamma counting study, we reduced the injected dose to allow more animals to be included in a single experiment with a single delivery of [^18^F]FLT. This was possible due to the high sensitivity of the Hidex gamma counter. Animals were recovered for 90 minutes and subsequently humanely culled by cervical dislocation under Schedule 1 of the UK (Scientific Procedures) Act 1986. Samples (blood, plasma, heart, spleen, kidneys, small and large intestines, supra-renal abdominal aorta, bone, and tail) were collected, weighed, and measured for radioactivity using a gamma counter (Hidex). Importantly, blood was removed from the aortic lumen before it was placed in the gamma counter. All ex vivo biodistribution data were decay-corrected, and radioactivity counts were expressed as the percentage of injected dose per gram of tissue (%ID/g).

### Ex Vivo Autoradiography

Following a 90-minute delay for biodistribution and gamma counting, spleen, control aorta, and 14-day AngII AAA whole-organ samples were placed on a phosphor screen (PerkinElmer) covered with clear cellophane wrap. After overnight exposure, the imaging plates were scanned using the Cyclone Plus imager (PerkinElmer) at a resolution of 300 DPI. Images were processed using OptiQuant software (PerkinElmer).

### Statistical Analysis

All statistical analyses were performed using Origin 2017 software (OriginLab Corporation, Northampton, MA, USA). All graphs show the mean ± the standard error of the mean (SE) unless otherwise stated. Histology, USS, and some gamma counting results were analyzed using two-sampled *t* tests with Welch’s correction. PET/CT, western blotting, and some gamma counting results were analyzed using one-way analysis of variance with post hoc Bonferroni-Holm correction. The threshold for statistical significance was set at *P *< 0.05.

## Results

### Cell Proliferation Occurs in AngII AAA

**Figure 1:**
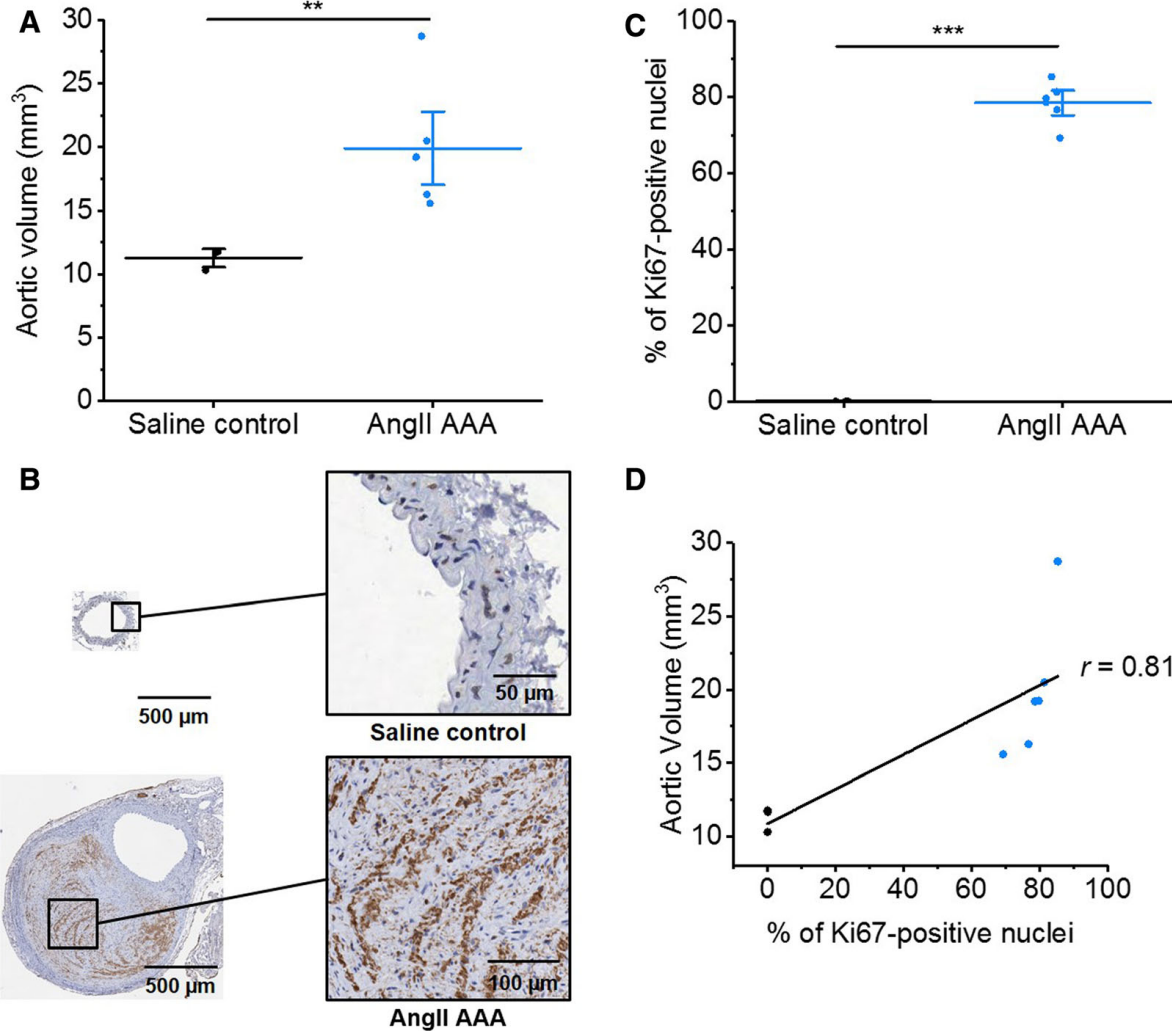
Ki67 is upregulated in 28-day AngII AAA tissue and correlated with aortic volume. (**A**) Aortic volumes in 28-day AngII AAA (*n *= 6) vs saline control aortae (*n *= 3). **P < 0.01 on two-sampled *t* test with Welch’s correction. (**B**) Immunohistochemical 3,3′-diaminobenzidine staining for Ki67 in AngII AAA (*n  *=  6) and saline control (*n  *=  3) aortae. Positive staining is brown. (**C**) Quantitative analysis of images in (**B**), % of Ki67-positive nuclei. ***P < 0.001 on two-sampled *t* test with Welch’s correction. (**D**) Correlation between aortic volume and Ki67-positive nuclei. Pearson’s *r  *=  0.81; black, saline control; blue, AngII AAA. AngII AAA, angiotensin II abdominal aortic aneurysm

As expected, ApoE^−/−^ mice infused with AngII developed significant supra-renal aortic lumen dilatation compared to the saline-infused controls (Figure 1A). To test for cell proliferation in AngII AAA, we stained fixed supra-renal abdominal aortic tissue from ApoE^−/−^ mice infused with saline or AngII for 28 days with Ki67, which accumulates in cells throughout the S, G2, and M cell cycle phases.^21^ A significant increase in the proportion of Ki67-positive nuclei was observed in the aortic wall in AngII-infused mice compared to saline controls (*P *< 0.001, Figure 1B to C), and this was positively correlated with aortic volume (Figure 1D).

### [^18^F]FLT Uptake and TK-1 Expression are Increased in AngII AAA

**Figure 2:**
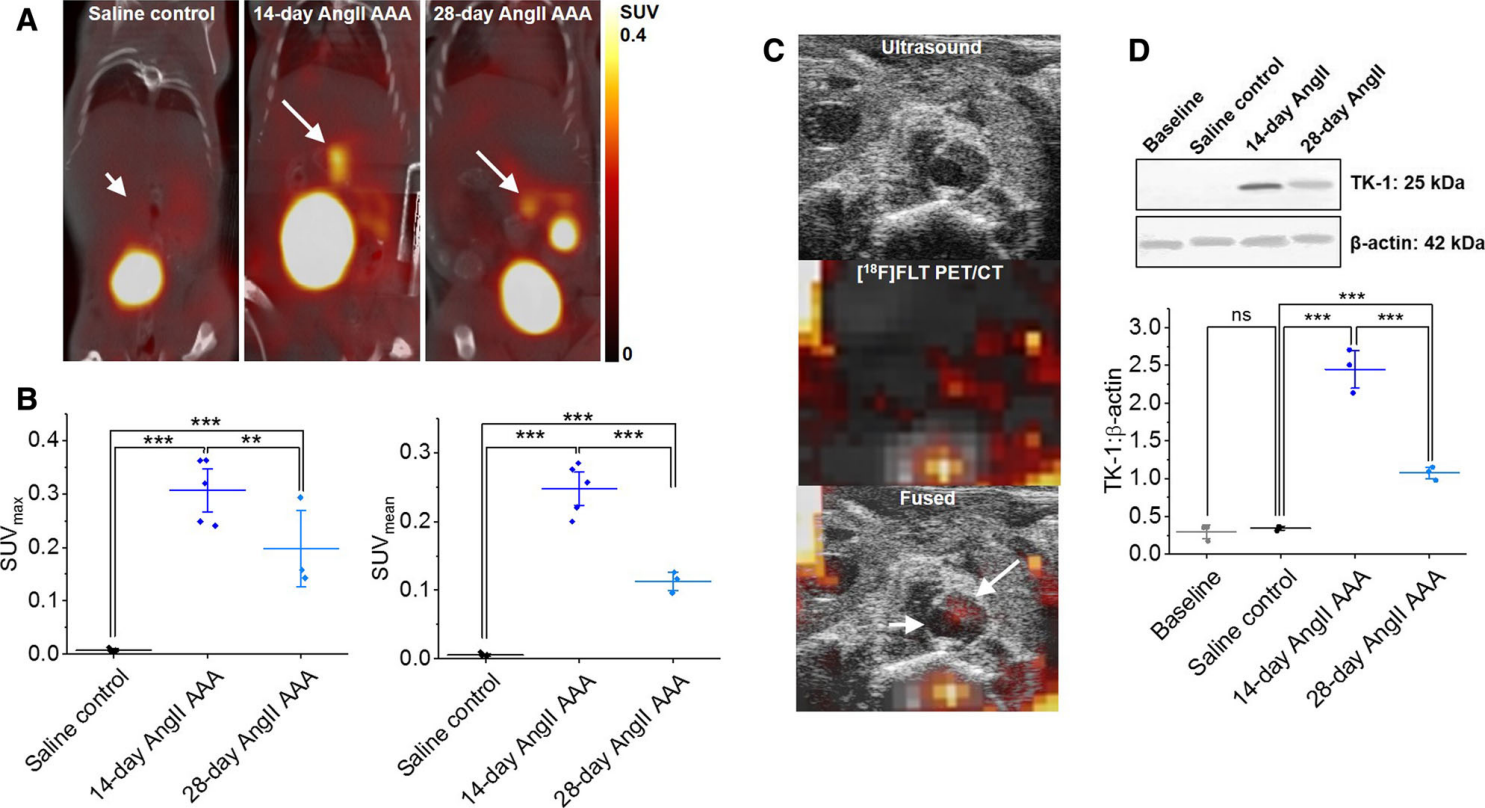
[^18^F]FLT uptake and TK-1 expression are greater in the 14-day AngII AAA model. (**A**) Representative coronal-view static [^18^F]FLT PET/CT images 80 to 90 minutes post-[^18^F]FLT injection. White arrows indicate abdominal aortic location (short arrow, saline control; long arrow, AAA). (**B**) Maximum and mean SUV analyses of abdominal aortic regions of interest on images in (**A**) of saline controls (*n *= 5), 14-day AngII AAA (*n *= 5), and 28-day AngII AAA (*n *= 3). (**C**) Representative transverse-view [^18^F]FLT PET/CT, ultrasound, and fused ultrasound-PET/CT images (short arrow, aortic lumen; long arrow, remodeling aortic wall). (**D**) Representative TK-1 and β-actin western blots and quantitative analysis, optical intensity of TK-1 band/optical intensity of β-actin band for baseline aortae (*n *= 3), saline control (*n *= 3), 14-day AngII AAA (*n *= 3), and 28-day AngII AAA (*n *= 3). ***P < 0.001, **P < 0.01, *ns*, not significant on one-way analysis of variance with post hoc Bonferroni-Holm correction. *[*^*18*^*F]FLT*, [^18^F]fluorothymidine; *AngII*, angiotensin II; *AAA*, abdominal aortic aneurysm; *PET/CT*, positron emission tomography/computed tomography; *SUV*, standardized uptake value; *TK-1*, thymidine kinase-1

Twenty-two mice were enrolled in the PET/CT study: five in the saline control group and seventeen in the AngII infusion group. Within the first 14 days of the study, ten mice died from aortic rupture in the AngII-infused group (8 thoracic, 2 abdominal), leaving twelve mice (5 saline controls and 7 AngII-infused) for imaging. A further four mice (2 saline controls and 2 AngII-infused) were excluded due to paravenous radiotracer injection. The PET/CT images obtained from the remaining 8 animals (3 saline controls and 5 AngII-infused) suggested increased [^18^F]FLT uptake in abdominal aortic ROIs (as shown in Supplementary Figure 1) after 14- and 28-day AngII infusions compared to saline controls. Notably, the signal was more intense after 14 days of AngII infusion, corresponding to the active growth phase in the AngII AAA model, than that after 28 days, corresponding to the end of the growth phase (Figure 2A; Supplementary Figure 3A to C, Supplementary Videos 1 to 4). It is noteworthy that repeat imaging of the AngII-infused mice on day 28 was limited to 3 animals as 1 injection was paravenous and 1 animal died from aortic rupture at the time of radiotracer injection. Splenic [^18^F]FLT uptake was observed consistently. Uptake was observed in the kidneys from 0.5 to 90 minutes and urinary bladder from 2 to 90 minutes after scan initiation, per its predominant renal clearance.

For quantification, SUV_max_ and mean SUVs (SUV_mean_) were used to construct time-activity curves for 0 to 90-minute aortic uptake post-[^18^F]FLT administration. Uptake in the saline control aortic ROIs remained consistently low throughout each scan. Meanwhile, all time-activity curves of the AngII AAA ROIs exhibited an initial peak corresponding to the bolus injection in the vena cava, followed by a plateau and late peak in the final 10 minutes of the scan (Supplementary Figure 2, Video 2). All subsequent SUV analyses were based on tracer uptake in the 80 to 90-minute timeframe.

Quantification and statistical analysis in all manually drawn ROIs revealed the following maximum SUVs (mean ± standard error of the mean): 0.007 ± 0.002, saline control; 0.31 ± 0.03, 14-day AngII AAA; 0.20 ± 0.05, 28-day AngII AAA. Significantly greater [^18^F]FLT uptake was observed in 14-day (*P *< 0.001) and 28-day (*P *< 0.001) AngII AAA compared to saline controls. The signal significantly decreased between 14- and 28-day AngII AAA (*P *< 0.01) (Figure 2B). Similar results were observed for SUV_mean_ (Figure 2B).

When USS was performed just before [^18^F]FLT PET/CT and the images were co-registered using bony landmarks, the observed PET signal appeared to overlay the remodeled and thickened aortic wall at the site of the aneurysm (Figure 2C).

Next, using an independent cohort of mice, we harvested the aortae at baseline and after saline and 14- and 28-day AngII infusions. We then extracted protein and performed western blotting for TK-1, the [^18^F]FLT substrate. We observed significantly increased TK-1 expression following 14-day AngII infusion compared to controls (*P *< 0.001) and significantly decreased TK-1 expression between 14- and 28-day AngII infusions (*P *< 0.001) (Figure 2D; uncropped blots in Supplementary Figure 4A), in keeping with the pattern of [^18^F]FLT activity observed during the PET/CT experiments.

To further confirm that the [^18^F]FLT PET/CT signal was reflective of proliferation, we treated AngII AAA mice with vehicle or the anti-proliferative agent imatinib by oral gavage for 3 days after the initial PET/CT scan and then repeated the imaging. The signal in the aortic ROI diminished after imatinib treatment but was unchanged following vehicle treatment (Supplementary Figure 5).

### Key [^18^F]FLT Transporter Proteins are Upregulated in AngII AAA

**Figure 3:**
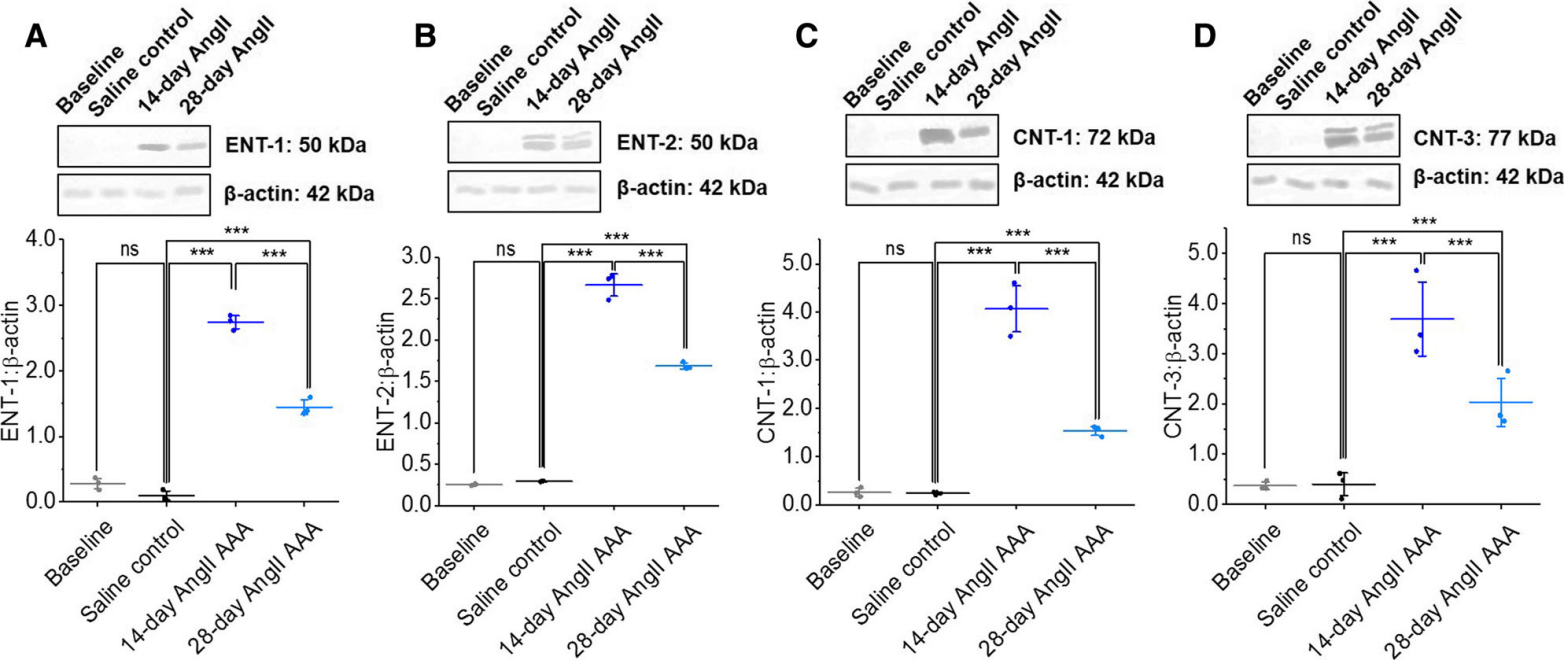
ENT-1, ENT-2, CNT-1, and CNT-3 expression is upregulated in 14-day AngII AAA tissue. Western blots for ENT-1 (**A**), ENT-2 (**B**), CNT-1 (**C**), and CNT-3 (**D**). All blots were repeated thrice in tissues from independent animals. The protein of interest was normalized to β-actin. ***P < 0.001, *ns*, not significant on one-way analysis of variance with post hoc Bonferroni-Holm correction. *[*^*18*^*F]FLT*, [^18^F]fluorothymidine; *ENT*, equilibrative nucleoside transporter; *CNT*, concentrative nucleoside transporter; *AngII AAA*, angiotensin II abdominal aortic aneurysm

Many [^18^F]FLT transporters are upregulated in cancer, aiding efficient [^18^F]FLT accumulation in proliferative cells.^22^,^23^ We observed significantly increased ENT-1, ENT-2, CNT-1, and CNT-3 expression in 14-day AngII AAA than in saline control aortae (*P *< 0.001 for all) and in 28-day AngII AAA (*P *< 0.001 for all). Any differences between baseline and saline control aortae were insignificant (Figure 3A to D; full uncropped blots in Supplementary Figure 4B to E).

### Aortic [^18^F]FLT Uptake is Correlated with Aortic Lumen Volume

**Figure 4:**
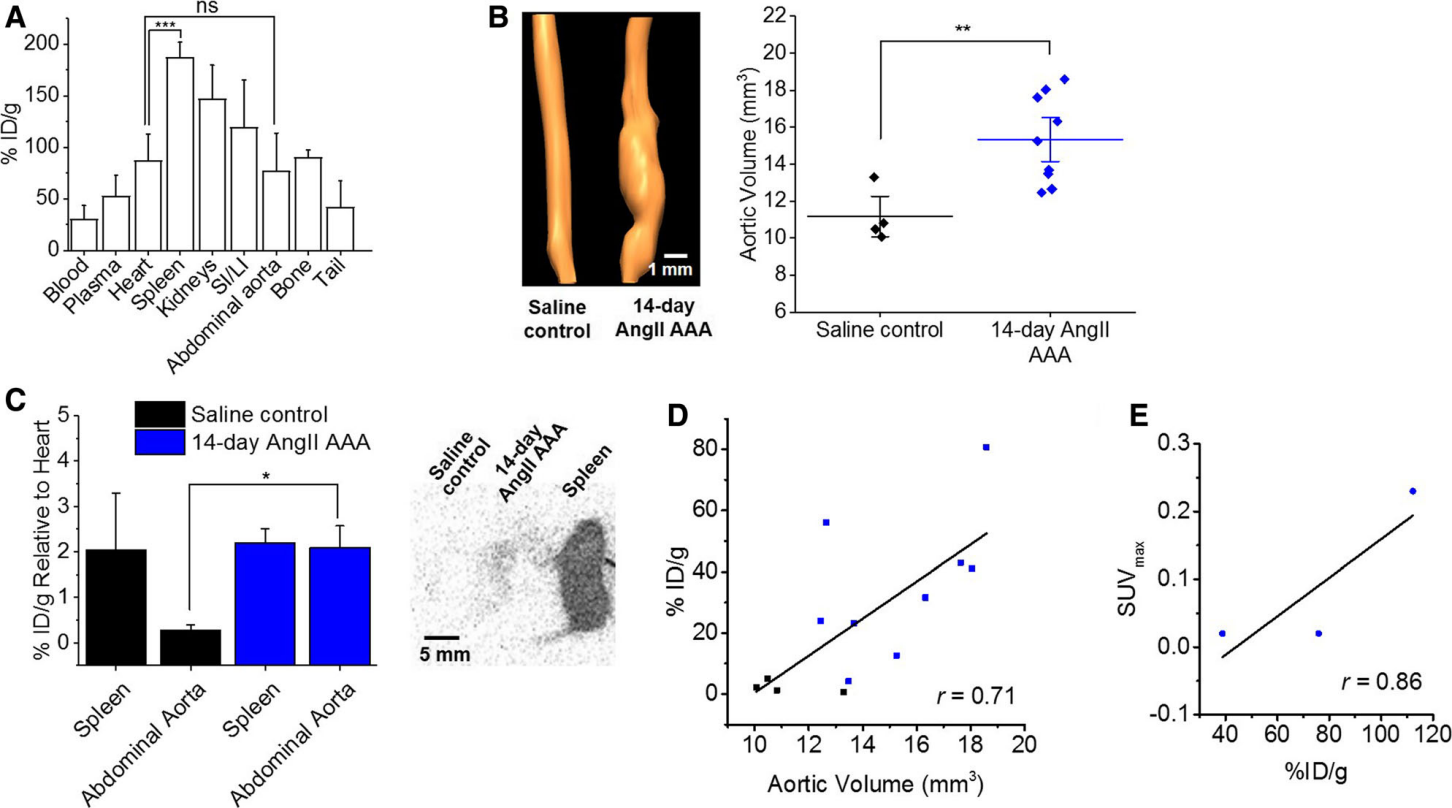
Aortic volume and [^18^F]FLT uptake are positively correlated. (**A**) Decay-corrected ex vivo [^18^F]FLT counts per mass units in baseline control C57BL6 mice (*n *= 4). Comparisons are shown for positive (spleen) vs negative (heart) controls and tissue of interest (aorta) vs heart. ***P < 0.001, *ns*, not significant on one-way analysis of variance with post hoc Bonferroni-Holm correction. (**B**) Representative USS three-dimensional aortic lumen reconstructions and volumes in 14-day AngII AAA (*n *= 9) vs saline control aortae (*n *= 4). **P < 0.01 on two-sampled *t* test with Welch’s correction. (**C**) Decay-corrected ex vivo [^18^F]FLT counts per mass units in spleens and abdominal aortae relative to the hearts from AngII AAA (*n *= 3) and saline control (*n *= 3) mice, and example ex vivo [^18^F]FLT autoradiography image using the same tissues. *P < 0.05 on two-sampled *t* test with Welch’s correction. The spleen is included as reference. (**D**) Correlation between aortic volume and ex vivo [^18^F]FLT counts per mass units. Pearson’s *r *= 0.71, *P *< 0.01. (**E**) Correlation between SUV_max_ and ex vivo [^18^F]FLT counts per mass units. Pearson’s *r *= 0.86; black, saline control; blue, AngII AAA. *USS*, ultrasound scanning; *AngII AAA*, angiotensin II abdominal aortic aneurysm; *[*^*18*^*F]FLT*, [^18^F]fluorothymidine; *SI/LI*, small and large intestines; *SUV*_*max*_, maximum standardized uptake value

As expected, baseline [^18^F]FLT biodistribution revealed high splenic uptake and low aortic and heart uptake. The aorta exhibited comparable uptake to the heart, while the spleen exhibited greater uptake relative to the heart (*P *= 0.69 vs *P *< 0.001) (Figure 4A); we therefore used the heart and spleen as negative and positive control tissues, respectively, for the AngII AAA study.

Three-dimensional USS demonstrated the expected aortic lumen volume increase following 14-day AngII infusion compared to saline-infused controls (15.34 ± 0.80 mm^3^ vs 11.16 ± 0.73 mm^3^, *P *< 0.01, Figure 4B). To confirm the origin of the aortic [^18^F]FLT signal, we performed ex vivo gamma counting of the aorta and spleen normalized to the heart following PET/CT (to control for mouse-to-mouse variability). We observed significantly increased [^18^F]FLT counts in aortic tissue from 14-day AngII-infused mice compared to saline controls (relative to the heart: *P *< 0.05, Figure 4C), consistent with our PET/CT and TK-1 western blotting data. The counts in 14-day AngII AAA were comparable to those observed in the spleen (%ID/g relative to the heart, mean ± standard error of the mean): 2.09 ± 0.32 and 2.19 ± 0.21, respectively. Ex vivo autoradiography further supported these data (Figure 4C). We observed a significant positive correlation between aortic volume and [^18^F]FLT uptake by gamma counting (Pearson’s *r *= 0.71, *P *< 0.01) (Figure 4D). The in vivo PET signal was also positively correlated with the ex vivo gamma counting signal when performed in the same animals (Figure 4E).

## Discussion

This is the first study to explore the feasibility of [^18^F]FLT PET/CT in an experimental murine model of progressive AAA. We demonstrated that [^18^F]FLT uptake in AngII-induced AAA increased after 14 days of infusion compared to saline controls with subsequent signal fall-off after 28 days. This was corroborated by a similar pattern of TK-1 protein expression in an independent cohort of animals. We confirmed that the [^18^F]FLT signal was originating from the aortic tissue by performing ex vivo gamma counting of supra-renal aortic tissue (without blood) and co-registered the PET/CT and USS data. Furthermore, ENT-1, ENT-2, CNT-1, and CNT-3 levels were observed to have increased in AngII AAA.

Initially, both [^18^F]FLT and thymidine are transported across the cell membrane via passive diffusion or specific carriers (ENT-1/2, CNT-1/3). The fluorine atom replacing the 3’ hydroxyl group prevents [^18^F]FLT from incorporating into DNA and proceeding along the thymidine mechanistic pathway. The cytosolic accumulation of phosphorylated [^18^F]FLT is detected using PET, reflecting TK-1 activity and thus proliferation.^24^,^25^ Phosphorylated [^18^F]FLT is unable to escape from cells and acts selectively as a substrate for TK-1, but not TK-2, contributing to the tracer’s specificity.^26^

[^18^F]FLT has been identified as a suitable PET radiotracer to assess proliferative activity in tumors.^24^,^27^,^28^ More recently, Ye *et al.* demonstrated [^18^F]FLT PET/CT uptake in atherosclerosis and a weak association between [^18^F]FLT and [^18^F]FDG uptake, suggesting that glucose metabolism and cell proliferation may be weakly associated in this context.^29^ Our results support the idea that [^18^F]FLT uptake might be useful in AAA as opposed to [^18^F]FDG uptake, for which the existing data are conflicting.

Selective [^18^F]FLT phosphorylation, and thus its uptake and retention in the cytosol, is limited by TK-1 during cell synthesis.^30^ It is therefore important that we observed significantly increased TK-1 expression after 14-day AngII infusion, when we observed the greatest [^18^F]FLT uptake on PET. TK-1 overexpression is similarly observed in cancer models,^30^,^31^ and its association with the Ki67 proliferation index has been demonstrated in various cancers.^32^-^34^ Interestingly, AAA [^18^F]FLT uptake was observed after 14 days of AngII infusion, when aneurysms expand, but this effect was weaker after 28 days, when the growth phase begins to plateau, suggesting decreased cell proliferation in late-stage AAA. This supports the idea of early-stage cellular remodeling leading to late-stage replicative senescence, which has been reported in end-stage human AAA samples.^17^,^35^,^36^ The early proliferative period may drive disease progression, but this theory requires further pre-clinical and clinical evaluation.

The nucleoside transporters that transport [^18^F]FLT into cells may also influence [^18^F]FLT uptake: ENT-1, ENT-2, CNT-1, and CNT-3. Paproski *et al.* characterized [^18^F]FLT transport by these transporters in cancer cells and demonstrated that they—particularly ENT-1 in murine tumor models—facilitate [^18^F]FLT uptake.^22^,^23^ Consistent with increased 14-day AngII AAA [^18^F]FLT uptake on in vivo PET/CT and ex vivo gamma counting, these transporters were significantly upregulated in 14-day AngII AAA tissues. The established association between [^18^F]FLT uptake and thymidine-associated marker expression in cancer is interesting to draw parallels with that in AAA. While concomitant malignancies occur in up to 14% of AAA cases 37 and smoking is an important shared risk factor between the two pathologies, we do not suggest a shared mechanism to the proliferation observed in our study in AAA and that previously reported in cancer. We merely observe that both pathologies involve cell proliferation.

Some limitations are associated with our study. The AngII AAA mouse model exhibits a high rate of mortality due to aneurysm rupture.^38^ Importantly, rupture in this model is not typically due to AAA progression, but is an early event in AAA initiation. Regarding PET/CT image analysis, the abdominal aorta and spleen are both anatomically near the bladder and kidneys, which readily take up [^18^F]FLT during excretion and consequently raise the possibility of signal spill-over into the aortic ROI. The ability to definitively study the aortic wall was also limited by the PET scanner’s spatial resolution (1 mm), making it difficult to evaluate tracer uptake in aneurysmal regions. To mitigate this risk, we used SUV_max_ for PET/CT analysis so that partial volume effects were minimal. We also took efforts to confirm that the PET/CT signal was originating from the aortic wall by conducting ex vivo gamma counting and performing co-registration of the 3D USS and PET datasets. Finally, the cell type(s) contributing to this proliferative [^18^F]FLT signal are insufficiently clear from our study, warranting further investigation.

## New Knowledge Gained

In 14-day AngII-induced AAA compared to 28-day AngII-induced AAA and saline controls, the [^18^F]FLT PET signal is significantly enhanced and [^18^F]FLT counts are greater, which themselves correlate with aortic volume. The expression of the [^18^F]FLT substrate, TK-1, and [^18^F]FLT transporter proteins ENT-1, ENT-2, CNT-1, and CNT-3 are also increased in AngII AAA. These findings suggest an early period of cell proliferation in the AngII AAA murine model, which is detectable using PET/CT.

## Conclusions

[^18^F]FLT is increased during the active growth phase of the AngII AAA murine model compared to saline control animals or late-stage AngII AAA. [^18^F]FLT uptake is correlated with aortic volume. The expression of the [^18^F]FLT substrate TK-1 is also increased in the model. Further work is necessary to determine the cell types contributing to the proliferative signal.

## Electronic supplementary material

Below is the link to the electronic supplementary material.

Supplementary material 1 (JPEG 189 kb)

Supplementary material 2 (JPEG 100 kb)

Supplementary material 3 (JPEG 231 kb)

Supplementary material 4 (JPEG 408 kb)

Supplementary material 5 (JPEG 85 kb)

Supplementary material 6 (MPG 867 kb)

Supplementary material 7 (MPG 411 kb)

Supplementary material 8 (MPG 443 kb)

Supplementary material 9 (MPG 227 kb)

Supplementary material 10 (DOCX 32 kb)

Supplementary material 11 (PPTX 615 kb)

Supplementary material 12 (M4A 3600 kb)

## Abbreviations

AAA: Abdominal aortic aneurysm
USS: Ultrasound scanning
PET: Positron emission tomography
CT: Computed tomography
[^18^F]FDG: Fluorine-18 fluorodeoxyglucose
[^18^F]FLT: Fluorine-18 fluorothymidine
ENT: Equilibrative nucleoside transporter
CNT: Concentrative nucleoside transporter
TK: Thymidine kinase
ApoE^−/−^: Apolipoprotein E-knockout
AngII: Angiotensin II
ROI: Region of interest
