# Supplementary figures and images for: Cell proliferation detected using [18F]FLT PET/CT as an early marker of abdominal aortic aneurysm
Source: J Nucl Cardiol. 2019 Nov 18;28(5):1961–71. doi: 10.1007/s12350-019-01946-y (PMC8648642; doi:10.1007/s12350-019-01946-y)

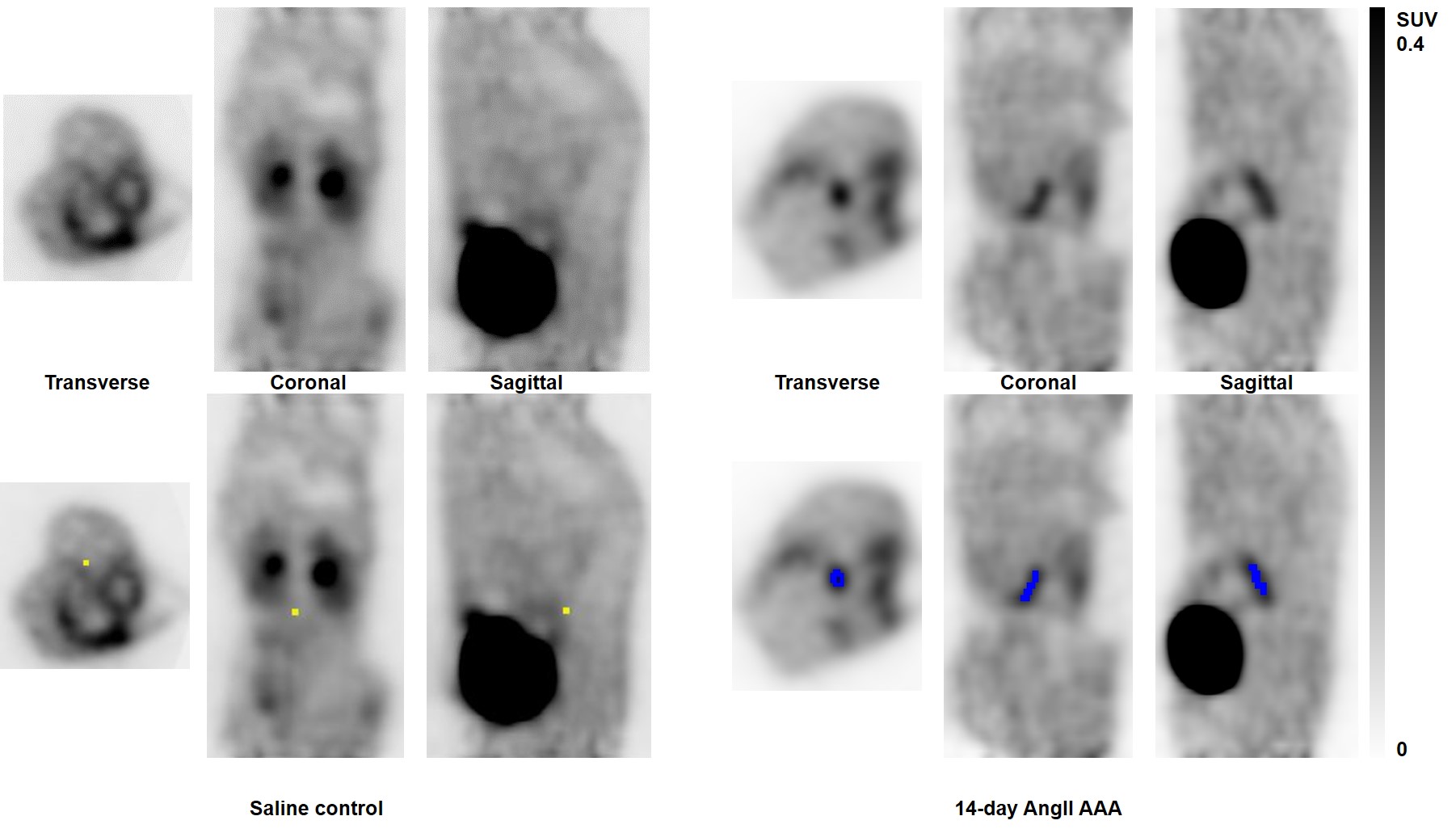

Supplement: Supplementary file 1 — Supplementary material 1 (JPEG 189 kb) [file 12350_2019_1946_MOESM1_ESM.jpg]

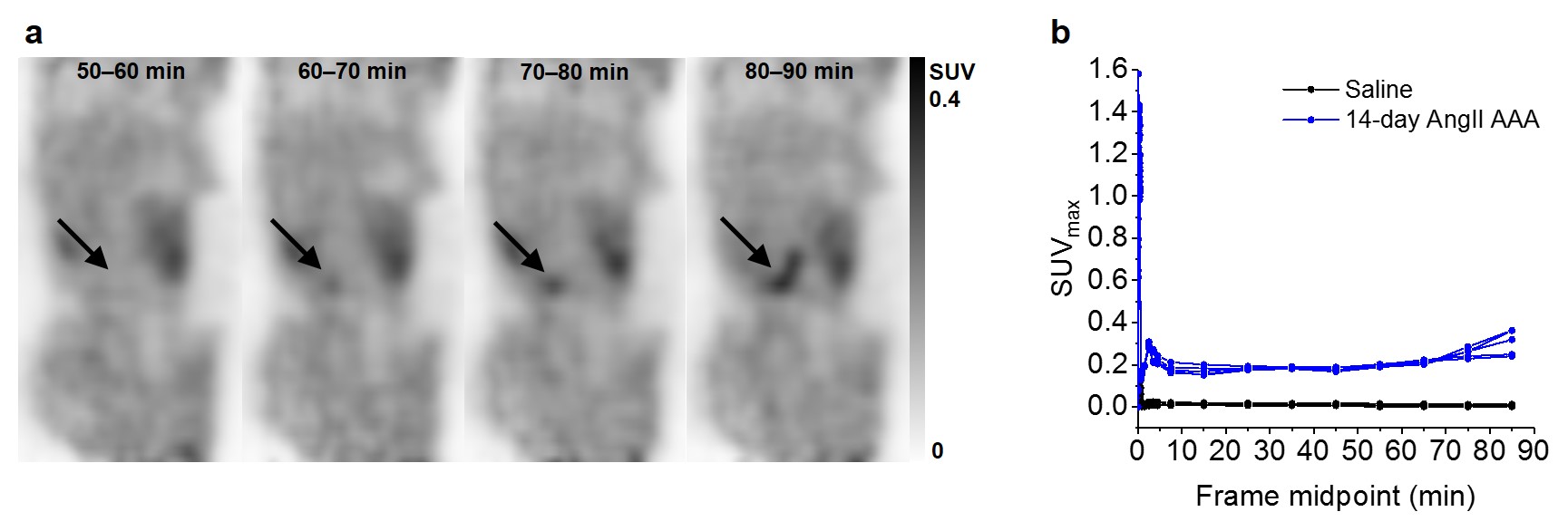

Supplement: Supplementary file 2 — Supplementary material 2 (JPEG 100 kb) [file 12350_2019_1946_MOESM2_ESM.jpg]

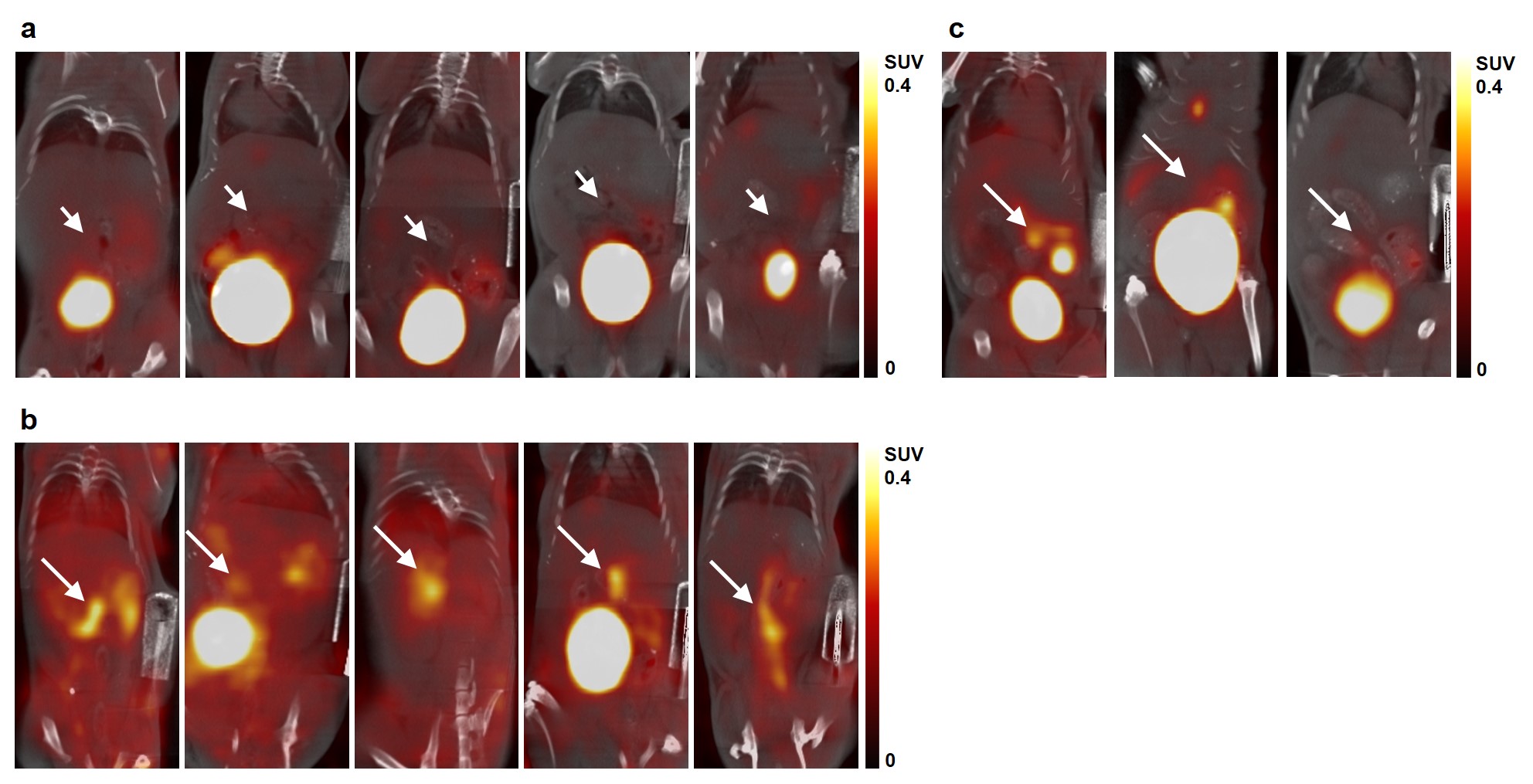

Supplement: Supplementary file 3 — Supplementary material 3 (JPEG 231 kb) [file 12350_2019_1946_MOESM3_ESM.jpg]

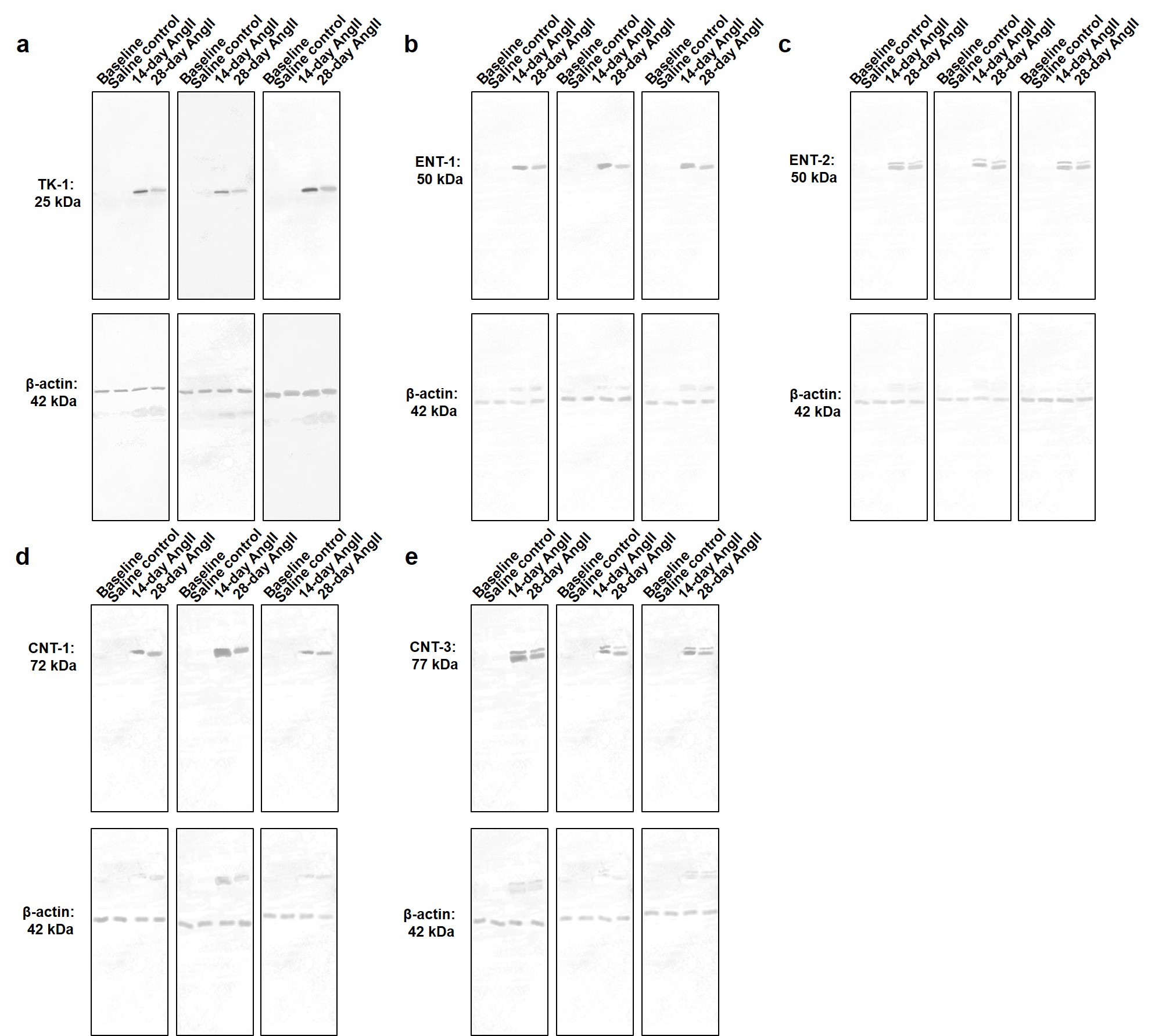

Supplement: Supplementary file 4 — Supplementary material 4 (JPEG 408 kb) [file 12350_2019_1946_MOESM4_ESM.jpg]

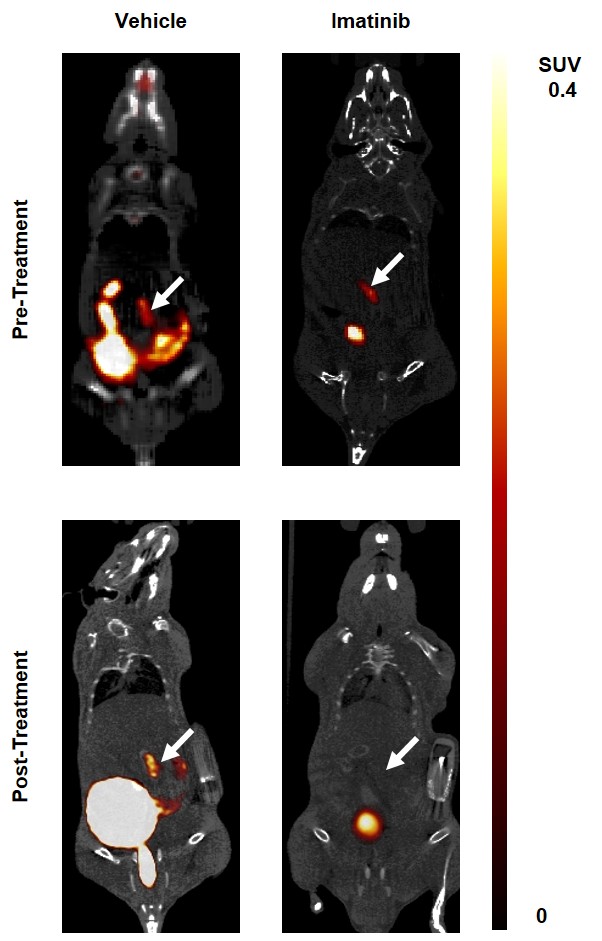

Supplement: Supplementary file 5 — Supplementary material 5 (JPEG 85 kb) [file 12350_2019_1946_MOESM5_ESM.jpg]
