# Supplementary material for: Cell proliferation detected using [18F]FLT PET/CT as an early marker of abdominal aortic aneurysm
Source: J Nucl Cardiol. 2019 Nov 18;28(5):1961–71. doi: 10.1007/s12350-019-01946-y (PMC8648642; doi:10.1007/s12350-019-01946-y)
Supplement: Supplementary file 10 — Supplementary material 10 (DOCX 32 kb) [file 12350_2019_1946_MOESM10_ESM.docx]

# Supplemental Legends

**Supplemental Fig. 1 Abdominal aortic ROIs for SUV analysis** Representative transverse-, coronal-, and sagittal-view static PET images and corresponding abdominal aortic ROI generation (yellow and blue shaded regions). ROI, region of interest; SUV, standardised uptake value; PET, positron emission tomography

**Supplemental Fig. 2 Dynamic [^18^F]FLT PET/CT images** a: Representative coronal-view dynamic images of 14-day AngII AAA model. Black arrows indicate abdominal aortic location. b: Time–activity curves based on abdominal aortic ROIs on [^18^F]FLT PET/CT images of 14-day AngII AAA and saline control mice. [^18^F]FLT, [^18^F]fluorothymidine; PET/CT, positron emission tomography/computed tomography; AngII AAA, angiotensin II abdominal aortic aneurysm; ROI, region of interest, SUV, standardised uptake value

**Supplemental Fig. 3 Complete [^18^F]FLT PET/CT images for the study** Representative images of all animal PET/CT data included in the study (coronal-view, static images at 80–90 min) of saline control mice (a), 14-day AngII AAA (b), and 28-day AngII AAA (c). White arrows indicate abdominal aortic location. [^18^F]FLT, [^18^F]fluorothymidine; AngII AAA, angiotensin II abdominal aortic aneurysm; PET/CT, positron emission tomography/computed tomography; SUV, standardised uptake value

**Supplemental Fig. 4 Full (uncropped) Western blot gels for Fig 2c and Fig 3a–d** a: TK-1 and β-actin. b: ENT-1 and β-actin. c: ENT-2 and β-actin. d: CNT-1 and β-actin. e: CNT-3 and β-actin. [^18^F]FLT, [^18^F]fluorothymidine; TK-1, thymidine kinase-1; ENT, equilibrative nucleoside transporter; CNT, concentrative nucleoside transporter; AngII AAA, angiotensin II abdominal aortic aneurysm.

**Supplemental Fig. 5 Pre- and post-treatment [^18^F]FLT PET/CT images** Representative coronal-view static PET/CT images of 14-day AngII AAA model pre- and post-treatment with vehicle or imatinib. White arrows indicate AAA location. [^18^F]FLT, [^18^F]fluorothymidine; PET/CT, positron emission tomography/computed tomography; AngII AAA, angiotensin II abdominal aortic aneurysm; SUV, standardised uptake value

**Supplemental Video 1. [^18^F]FLT PET/CT flythrough** Representative coronal-view dynamic PET/CT flythrough of 14-day AngII AAA model, progressing anteriorly from the vertebrae. [^18^F]FLT signal is noted in the kidneys, AAA, spleen, and bladder. [^18^F]FLT, [^18^F]fluorothymidine; PET/CT, positron emission tomography/computed tomography; AngII, angiotensin II; AAA, abdominal aortic aneurysm

**Supplemental Video 2. Dynamic uptake of [^18^F]FLT** Representative coronal-view dynamic PET/CT time-lapse video of 14-day AngII AAA model 10–90 min post-injection of [^18^F]FLT. Gradual signal in the AAA is noted. [^18^F]FLT, [^18^F]fluorothymidine; PET/CT, positron emission tomography/computed tomography; AngII, angiotensin II; AAA, abdominal aortic aneurysm

**Supplemental Video 3. [^18^F]FLT PET/CT flythrough** Representative coronal-view dynamic PET/CT flythrough of 14-day saline control, progressing anteriorly from the vertebrae. [^18^F]FLT signal is noted in the kidneys, bladder, and spleen. [^18^F]FLT, [^18^F]fluorothymidine; PET/CT, positron emission tomography/computed tomography

**Supplemental Video 4. Dynamic uptake of [^18^F]FLT** Representative coronal-view dynamic PET/CT time-lapse video of 14-day saline control 10–90 min post-injection of [^18^F]FLT. Signal in the kidneys is noted. [^18^F]FLT, [^18^F]fluorothymidine; PET/CT, positron emission tomography/computed tomography
