# Supplementary material for: Cell proliferation detected using [18F]FLT PET/CT as an early marker of abdominal aortic aneurysm
Source: J Nucl Cardiol. 2019 Nov 18;28(5):1961–71. doi: 10.1007/s12350-019-01946-y (PMC8648642; doi:10.1007/s12350-019-01946-y)
Supplement: Supplementary file 11 — Supplementary material 11 (PPTX 615 kb) [file 12350_2019_1946_MOESM11_ESM.pptx]

## Slide 1
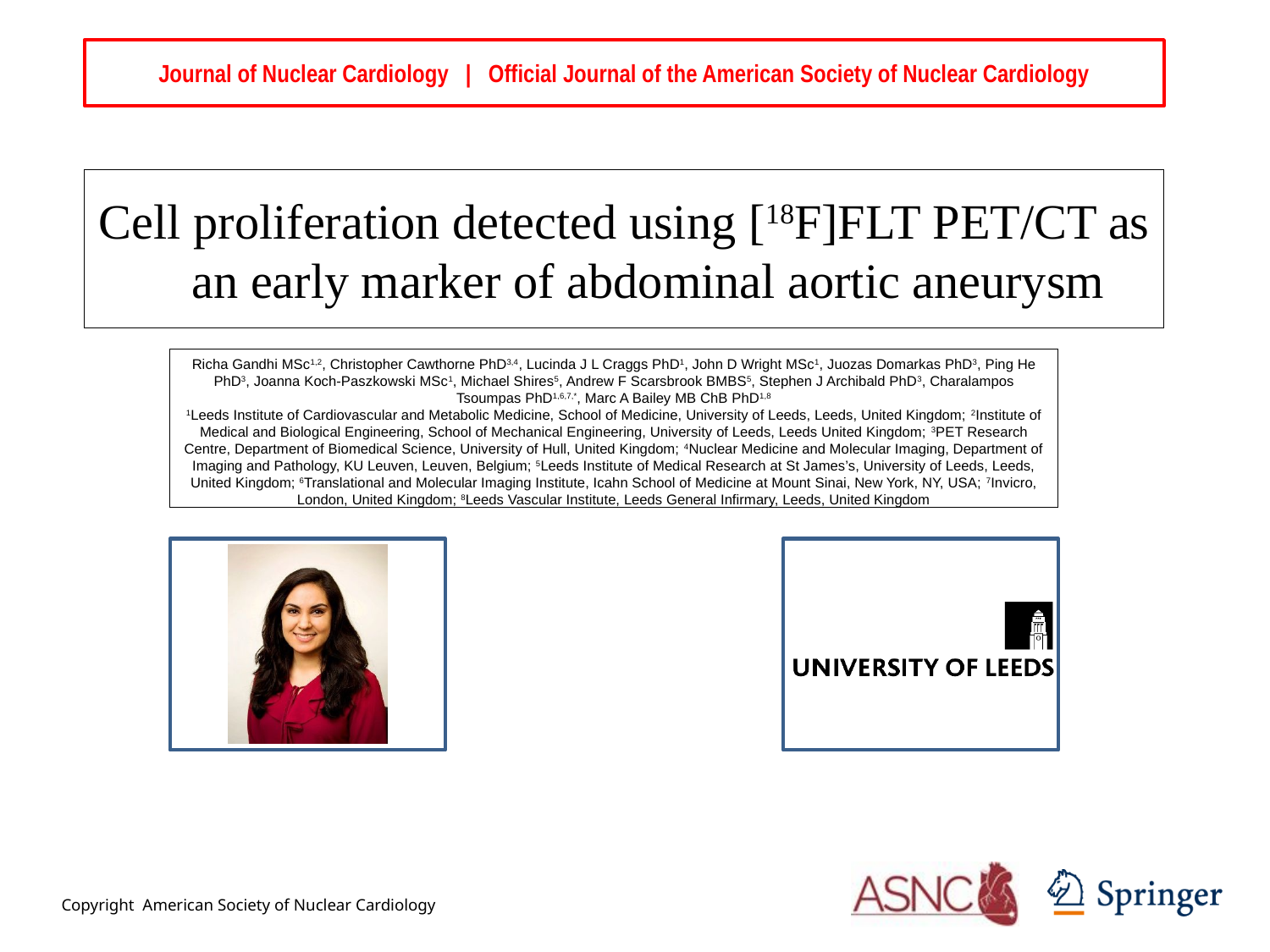

Journal of Nuclear Cardiology | Official Journal of the American Society of Nuclear Cardiology
# Cell proliferation detected using [18F]FLT PET/CT as an early marker of abdominal aortic aneurysm
Richa Gandhi MSc1,2, Christopher Cawthorne PhD3,4, Lucinda J L Craggs PhD1, John D Wright MSc1, Juozas Domarkas PhD3, Ping He PhD3, Joanna Koch-Paszkowski MSc1, Michael Shires5, Andrew F Scarsbrook BMBS5, Stephen J Archibald PhD3, Charalampos Tsoumpas PhD1,6,7,*, Marc A Bailey MB ChB PhD1,8
1Leeds Institute of Cardiovascular and Metabolic Medicine, School of Medicine, University of Leeds, Leeds, United Kingdom; 2Institute of Medical and Biological Engineering, School of Mechanical Engineering, University of Leeds, Leeds United Kingdom; 3PET Research Centre, Department of Biomedical Science, University of Hull, United Kingdom; 4Nuclear Medicine and Molecular Imaging, Department of Imaging and Pathology, KU Leuven, Leuven, Belgium; 5Leeds Institute of Medical Research at St James’s, University of Leeds, Leeds, United Kingdom; 6Translational and Molecular Imaging Institute, Icahn School of Medicine at Mount Sinai, New York, NY, USA; 7Invicro, London, United Kingdom; 8Leeds Vascular Institute, Leeds General Infirmary, Leeds, United Kingdom
Copyright American Society of Nuclear Cardiology

## Slide 2
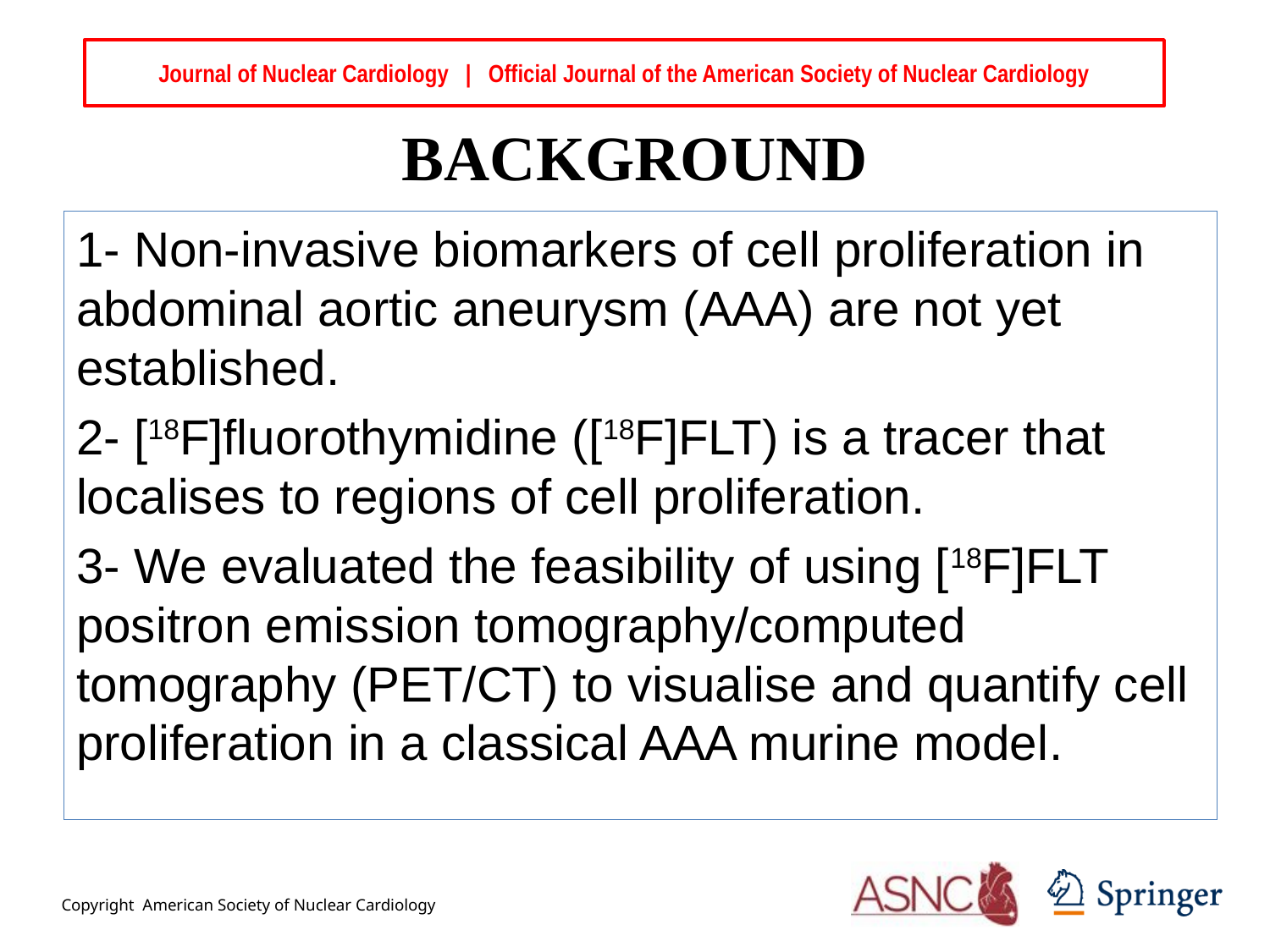

Journal of Nuclear Cardiology | Official Journal of the American Society of Nuclear Cardiology
# BACKGROUND
1- Non-invasive biomarkers of cell proliferation in abdominal aortic aneurysm (AAA) are not yet established.
2- [18F]fluorothymidine ([18F]FLT) is a tracer that localises to regions of cell proliferation.
3- We evaluated the feasibility of using [18F]FLT positron emission tomography/computed tomography (PET/CT) to visualise and quantify cell proliferation in a classical AAA murine model.
Copyright American Society of Nuclear Cardiology

## Slide 3
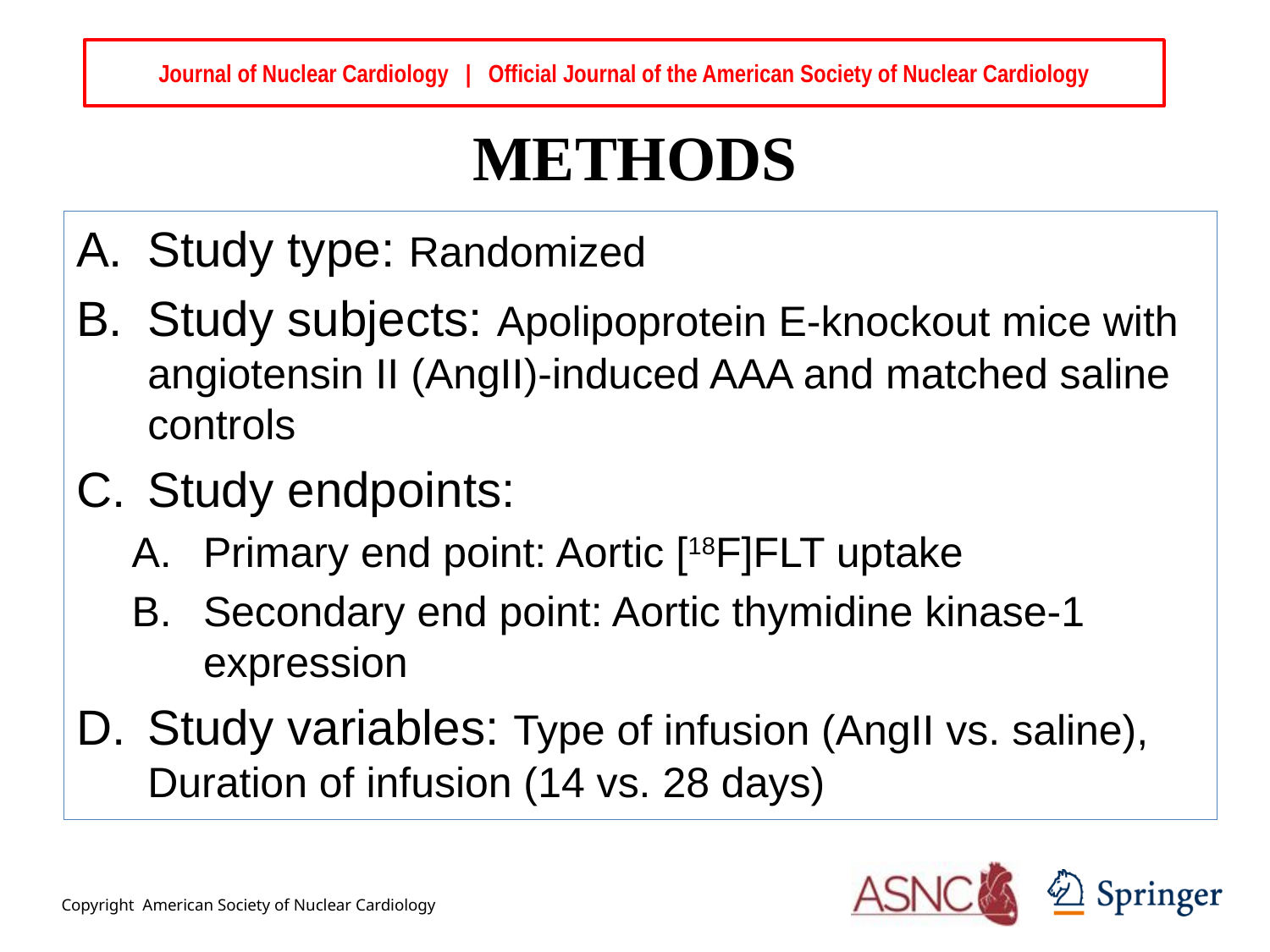

Journal of Nuclear Cardiology | Official Journal of the American Society of Nuclear Cardiology
# METHODS
Study type: Randomized
Study subjects: Apolipoprotein E-knockout mice with angiotensin II (AngII)-induced AAA and matched saline controls
Study endpoints:
Primary end point: Aortic [18F]FLT uptake
Secondary end point: Aortic thymidine kinase-1 expression
Study variables: Type of infusion (AngII vs. saline), Duration of infusion (14 vs. 28 days)
Copyright American Society of Nuclear Cardiology

## Slide 4
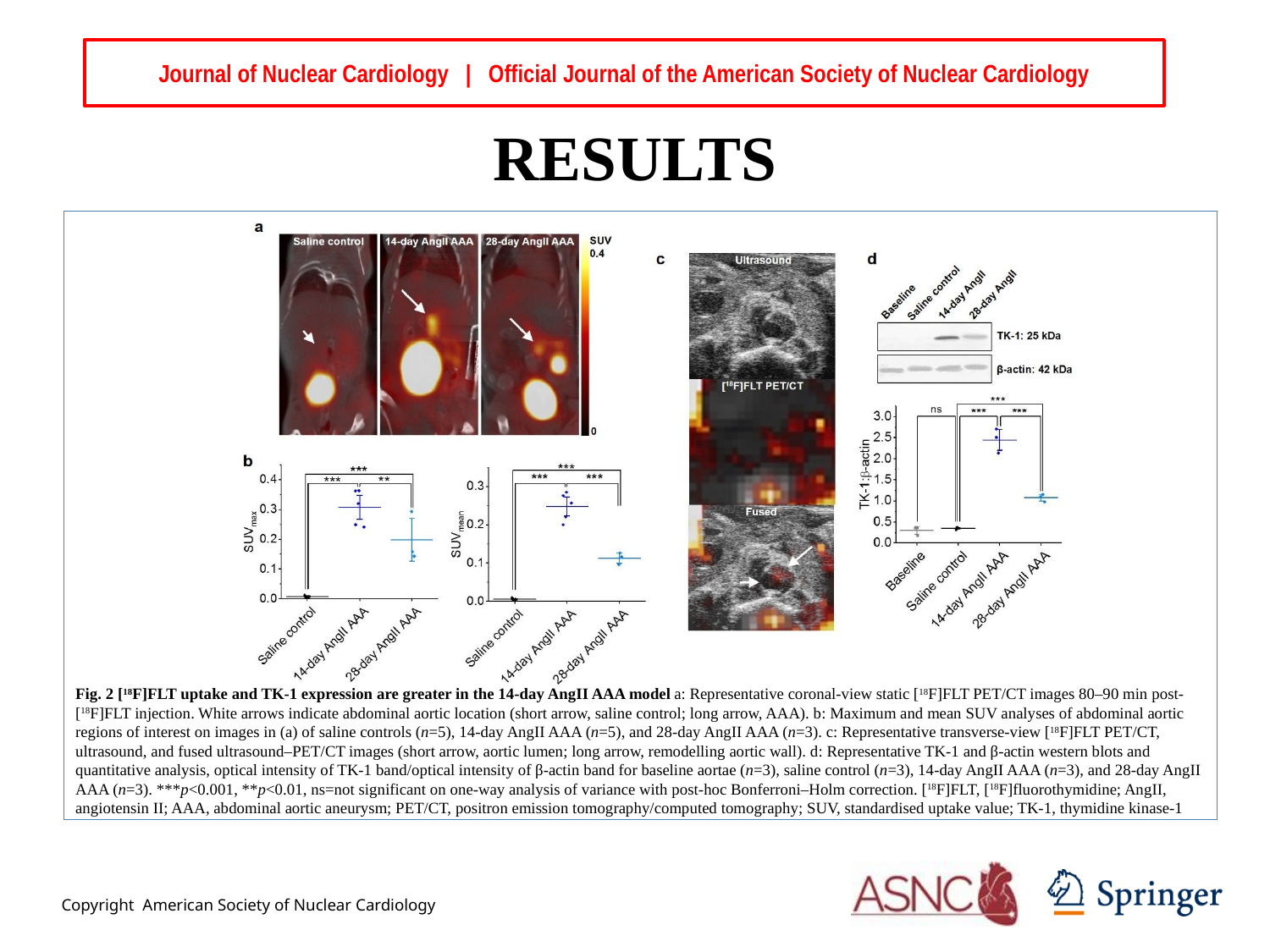

Journal of Nuclear Cardiology | Official Journal of the American Society of Nuclear Cardiology
# RESULTS
Fig. 2 [18F]FLT uptake and TK-1 expression are greater in the 14-day AngII AAA model a: Representative coronal-view static [18F]FLT PET/CT images 80–90 min post-[18F]FLT injection. White arrows indicate abdominal aortic location (short arrow, saline control; long arrow, AAA). b: Maximum and mean SUV analyses of abdominal aortic regions of interest on images in (a) of saline controls (n=5), 14-day AngII AAA (n=5), and 28-day AngII AAA (n=3). c: Representative transverse-view [18F]FLT PET/CT, ultrasound, and fused ultrasound–PET/CT images (short arrow, aortic lumen; long arrow, remodelling aortic wall). d: Representative TK-1 and β-actin western blots and quantitative analysis, optical intensity of TK-1 band/optical intensity of β-actin band for baseline aortae (n=3), saline control (n=3), 14-day AngII AAA (n=3), and 28-day AngII AAA (n=3). ***p<0.001, **p<0.01, ns=not significant on one-way analysis of variance with post-hoc Bonferroni–Holm correction. [18F]FLT, [18F]fluorothymidine; AngII, angiotensin II; AAA, abdominal aortic aneurysm; PET/CT, positron emission tomography/computed tomography; SUV, standardised uptake value; TK-1, thymidine kinase-1
Copyright American Society of Nuclear Cardiology

## Slide 5
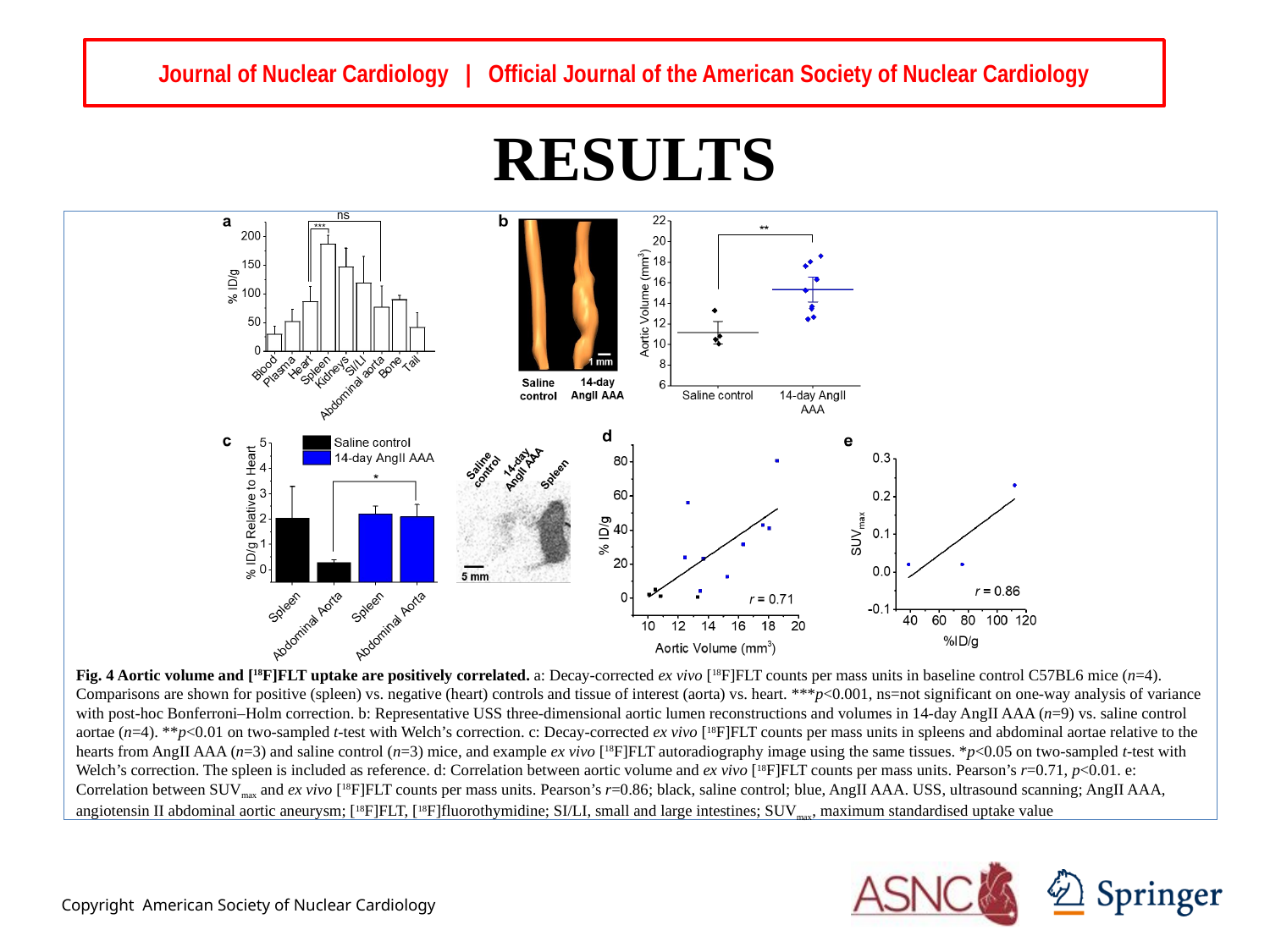

Journal of Nuclear Cardiology | Official Journal of the American Society of Nuclear Cardiology
# RESULTS
Fig. 4 Aortic volume and [18F]FLT uptake are positively correlated. a: Decay-corrected ex vivo [18F]FLT counts per mass units in baseline control C57BL6 mice (n=4). Comparisons are shown for positive (spleen) vs. negative (heart) controls and tissue of interest (aorta) vs. heart. ***p<0.001, ns=not significant on one-way analysis of variance with post-hoc Bonferroni–Holm correction. b: Representative USS three-dimensional aortic lumen reconstructions and volumes in 14-day AngII AAA (n=9) vs. saline control aortae (n=4). **p<0.01 on two-sampled t-test with Welch’s correction. c: Decay-corrected ex vivo [18F]FLT counts per mass units in spleens and abdominal aortae relative to the hearts from AngII AAA (n=3) and saline control (n=3) mice, and example ex vivo [18F]FLT autoradiography image using the same tissues. *p<0.05 on two-sampled t-test with Welch’s correction. The spleen is included as reference. d: Correlation between aortic volume and ex vivo [18F]FLT counts per mass units. Pearson’s r=0.71, p<0.01. e: Correlation between SUVmax and ex vivo [18F]FLT counts per mass units. Pearson’s r=0.86; black, saline control; blue, AngII AAA. USS, ultrasound scanning; AngII AAA, angiotensin II abdominal aortic aneurysm; [18F]FLT, [18F]fluorothymidine; SI/LI, small and large intestines; SUVmax, maximum standardised uptake value
Copyright American Society of Nuclear Cardiology

## Slide 6
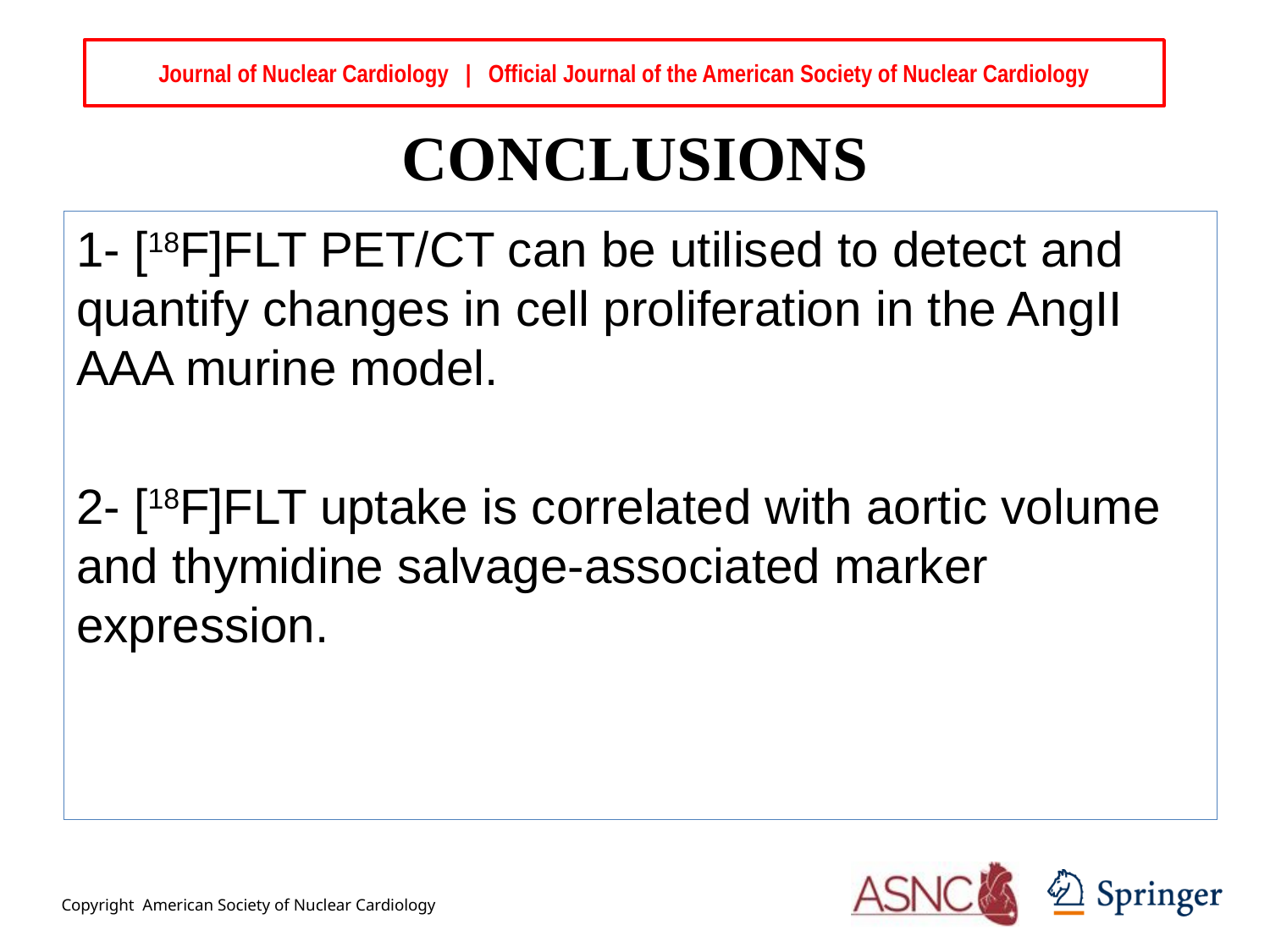

Journal of Nuclear Cardiology | Official Journal of the American Society of Nuclear Cardiology
# CONCLUSIONS
1- [18F]FLT PET/CT can be utilised to detect and quantify changes in cell proliferation in the AngII AAA murine model.
2- [18F]FLT uptake is correlated with aortic volume and thymidine salvage-associated marker expression.
Copyright American Society of Nuclear Cardiology
